# Editorial: Genetics and genomics of plant reproduction for crop breeding, volume II

**DOI:** 10.3389/fpls.2023.1145208

**Published:** 2023-02-14

**Authors:** Gianni Barcaccia, Andrea Mazzucato, Emidio Albertini, Sara Zenoni, Luciana Baldoni, Soraya Mousavi, Marta Adelina Mendes, Silvia Coimbra, Antonio Granell, Fulvio Pupilli

**Affiliations:** ^1^ Department of Agronomy Food Natural resources Animals and Environment (DAFNAE), University of Padova, Padova, Italy; ^2^ Department of Agriculture and Forest Sciences (DAFNE), University of Tuscia, Viterbo, Italy; ^3^ Department of Agricultural, Food and Environmental Sciences (DSA3), University of Perugia, Perugia, Italy; ^4^ Department of Biotechnologies, University of Verona, Verona, Italy; ^5^ Institute of Biosciences and Bioresources, Research Division of Perugia, National Research Council (CNR), Perugia, Italy; ^6^ Department of Biosciences, University of Milan, Milan, Italy; ^7^ Department of Biology, Faculty of Sciences, University of Porto, Porto, Portugal; ^8^ Institute of Molecular and Cellular Biology of Plants, Spanish National Research Council (CSIC), Polytechnic University of Valencia, Valencia, Spain

**Keywords:** apomixis, apomeiosis, parthenogenesis, male-sterility, self-incompatibility, parthenocarpy, polyploidy, plant breeding

The main challenges of modern agriculture deal with sustainably producing food and raw materials while contributing to environment preservation and mitigation of climate change risks worldwide. The actual unprecedented global economic and social crisis due to the Coronavirus pandemic, along with the current geo-politics instability due to Russia’s war in Ukraine and the modified climate conditions, will likely mean a turning point to deal with the overall resilience of agriculture systems and sustainability of food supplies (Barcaccia et al., 2020).

In a not too far coming future, increasing the production per hectare of cultivated plants will be necessary to cope with the increasing human population and the gradual deterioration of environmental conditions, especially in developing countries. Suppose it is true that next-generation genotyping and phenotyping platforms enable to predict and select resistance to plant pathogens and tolerance to environmental stresses. In that case, it is also true that new breeding techniques allow the developing of improved varieties based on superior genotypes enriched or edited for single genes/traits so to ensure greater unit yields (“more with less” principle) and better quality for economically important plant species (“do no significant harm” principle). In this context, studying and understanding the mechanisms that regulate reproductive systems is crucial as, in many cases, the stability and yield of crop plants depend on genetic factors and networks controlling fundamental aspects of seed and fruit development.

Reproduction in angiosperms has a well-defined evolutionary meaning. If, on the one hand, it markedly determines the preservation of genetic diversity between species, limiting or avoiding an exchange of genes between different genomes, on the other hand, it equally affects the genetic structure of populations, significantly contributing to the composition of genotypes within species and the organization of their genomes (**Figure 1**). Consequently, the opportunities and modalities for the genetic improvement of crop populations and the development of new varieties depend on plant reproductive systems and barriers, including male-sterility, self-incompatibility, parthenocarpy, and apomeiosis/parthenogenesis or apomixis. In a general view, comprehending the molecular basis of the reproductive systems of crop plants will help overcome the reproductive barriers that limit crop yield and quality, and serve to understand the evolution and structure of plant populations.

**Figure 1:**
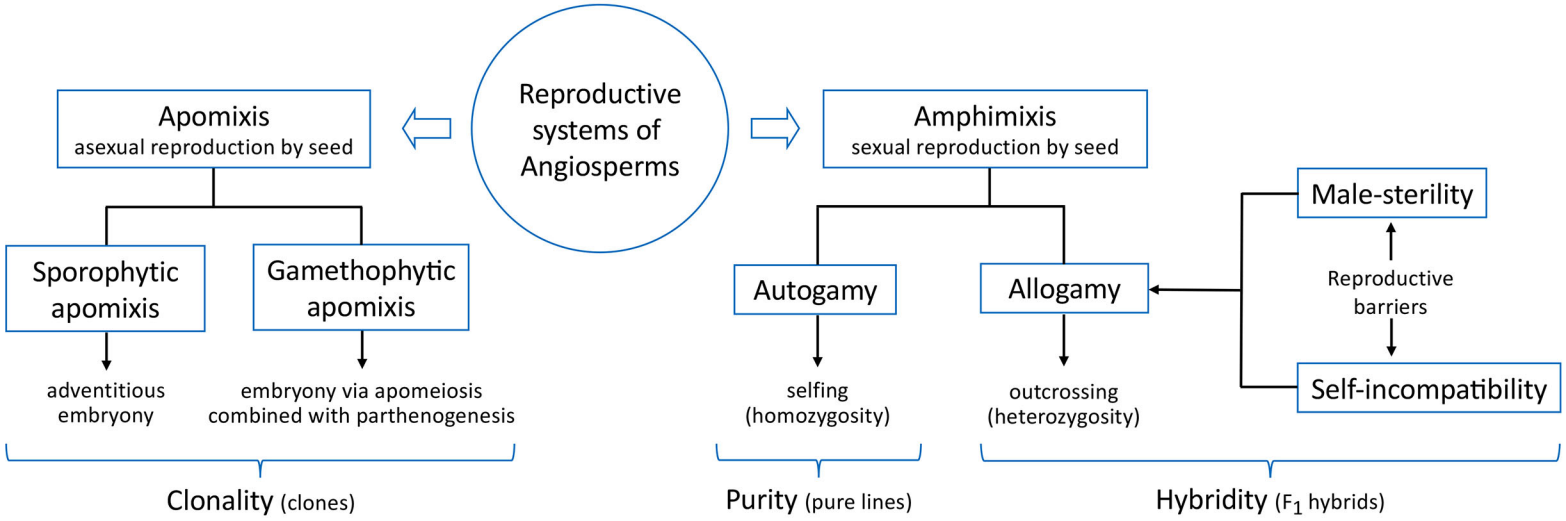
Main reproductive systems and barriers in crop plants: apomixis determines clonal populations by means of either sporophytic embryony or gamethophytic embryony, whereas amphimixis through selfing and outcrossing is exploited for breeding pure lines and F1 hybrids, respectively.

Our Research Topic, Genetics and Genomics of Plant Reproduction for Crop Breeding, includes a total of 25 articles in its first volume (Research Topic, vol. 1) and additional 12 articles in its second volume (Research Topic, vol. 2). It represents a valuable collection of both Original Research and Review Articles focused on the recent advances of our knowledge on the genetics of crop plant reproductive systems owing to the application and exploitation of novel biotechnological tools supported by information deriving from modern genomics. Several articles provided insights on identifying the genetic mechanisms and factors that control the expression of sexual barriers and molecular markers for their prediction in important horticultural crops and fruit trees. The latter include male-sterility in leaf chicory, tomato, and foxtail millet (Palumbo et al.; Takei et al.; Gao et al.), self-incompatibility in cabbage, pear, and olive (Alagna et al.; Claessen et al.; Mariotti et al.; Xiao et al.) and parthenocarpy in tomato and, more in general, in higher plants (Picarella and Mazzucato; Takei et al.). Other articles reported consistent findings from the identification and/or functional analysis of candidate genes for apomixis and its components (apomeiosis, i.e., apospory or diplospory, parthenogenesis, and autonomous endosperm development) in apomictic model species (Mancini et al.; Galla et al.; Kaushal et al.; Vijverberg et al.; Zappacosta et al.; Cielo Pasten et al.; Singh Sidhu et al.; Xia et al.). Worth of mentioning several very interesting review articles on the topics of apomixis as a whole (i.e., The rise of apomixis in natural plant populations by Hojsgaard and Hörandl; Partitioning apomixis components to understand and utilize gametophytic apomixis by Kaushal et al.; Does Polyploidy inhibit sex chromosome evolution in Angiosperms? By He and Hörandl; Trends in apomixis research: the ten most cited research articles published in the pregenomic and genomic eras by Palumbo et al.), parthenogenesis (i.e., Identifying and engineering genes for parthenogenesis in plants by Vijverberg, Ozias-Akins, and Schranz), self-incompatibility (i.e., Finding a compatible partner: molecular control, genetic determination, and impact on fertilization and fruit set of self-incompatibility in European pear by Claessen et al.; The paradox of self-fertile varieties in the context of self-incompatible genotypes in olive by Alagna et al.) and parthenocarpy (i.e., The occurrence of seedlessness in higher plants, with insights on roles and mechanisms of parthenocarpy by Picarella and Mazzucato, 2019).

The potential use of new biotechnological approaches for either loss-of-function and gain-of-function applications, such as cisgenesis (for conspecific gene transfer or introgression) and genome editing (for endogenous gene knockout or silencing and gene editing or replacement), will assume strong relevance in the future of agriculture, as these methods would allow direct intervention at the genomic level in any variety without changing its genetic background (Pirrello et al., 2022). All these genome-wide biotechnological strategies contribute to the achievement of new rapidly increasing precision breeding methods that are potentially useful for developing cultivars genetically improved for single traits while preserving the rest of the genome. In addition, these methods reduce the time needed to fix a superior genotype in an elite cultivar, avoiding any genetic recombination or transfer of unwanted genetic material. Furthermore, in the coming years, it seems possible that biotechnological approaches could allow not only cisgenesis transfer or the editing of genes of interest but also the control of whole biosynthetic pathways and regulatory networks, making the improvement of crop plant cultivars for complex agronomic traits achievable by intervening in the development or composition of specific tissues and organs.

It is well known that the possibility to control fertilization to achieve full hybridization between genetically divergent parental lines is highly desirable for breeding F1 hybrid seeds and heterotic varieties, as farmers demand. Natural parthenocarpy is also desirable as a strategy for producing seedless fruits, increasingly appreciated by consumers. With the strengthening of genomics, mapping and cloning the genes that control meiosis, gametogenesis, pollen-pistil interaction, and fertilization-independent hybrid seed and seedless fruit production is at our fingertips. For the most important crop plants, traditional breeding approaches have been used extensively to develop new cultivars with desirable characteristics, including resistance/tolerance to biotic and abiotic stresses, high yield, and a high content of compounds beneficial to human health. The technological progress of the last few decades has revolutionized our ability to study and manipulate genetic variation in crop plants. The development of high-throughput sequencing platforms and accompanying analytical methods have led to the sequencing and assembling of a large number of plant genomes, the construction of dense and ultra-dense molecular linkage maps, the identification of structural variants, and the application of molecular markers in breeding programs (Reviewed by Simko et al., 2021).

We are confident that the control of plant reproduction systems and barriers can potentially change the distribution and scale of investments for breeding new varieties, disrupt existing commercial supply chains and lead to greater uptake and use of agronomic sources and nutritional traits, contributing so to environmental quality traits and mitigation of climate change risks. We expect that genomics and biotechnologies can play a central role in modern plant breeding, as their main applicative tools and platforms are capable of making a pivotal contribution to renewing the varietal landscape for the main crops and restarting the whole primary sector and agri-food industry, also addressing social and environmental issues, and so accelerating the transition to sustainability (Barcaccia et al., 2020).

As technologies to overcome sexual barriers, as others related to genetic improvement of crops are mainly focused on guaranteeing sufficient, safe, and nutrient food worldwide, efforts in research and investments should be maintained under governmental control.

## Author contributions

GB: conceptualization. GB: writing—original draft preparation. All authors listed have made a substantial, direct, and intellectual contribution to review and edit this version of the work and approved it for publication.
